# Supplementary material for: Multifiber Array‐Based Photometry System for Multiregional Functional Mapping in the Mouse Brain
Source: Eur J Neurosci. 2026 Jun 17;63(12):e70582. doi: 10.1111/ejn.70582 (PMC13273707; doi:10.1111/ejn.70582)
Supplement: Supplementary file 1 — Table S1: Optical system parts list. Table S2: Surgical parameters. Table S3: Parameters for data analysis. Figure S1: Light power output estimation. (A) Diagram of an empirical approach to light output estimation. (B) A linear formula to estimate light output and the resultant tables. The light power from the objective lens was 69.3 mW. (C) The relationship between the modelled light power profiles and the distance from the illumination center. The values were calculated based on a quality control image (Figure 2D). Pearson's r = −0.65, p < 0.005. Figure S2: The effect of preprocessing before motion correction on 405‐nm images. Correlation to the reference was compared between two conditions, with and without a high‐pass (HP) filter. Inset, Correlation of variation in pixel intensity. ***p < 0.001, Wilcoxon signed‐rank test. Figure S3: Noise budget analysis. (A) Mean fluorescent signals in e− across active fibers and experiments. (B) High‐frequency (HF) noise floor across active fibers and experiments. The HF noise floor was computed using successive frame differences as std (ΔF)/√2, where ΔF is the first difference in the photoelectron time series. The HF noise floor was expressed in ΔF/F by normalizing them to the mean fluorescence. The total theoretical noise (dotted line) was estimated by estimating photon shot noise and camera read/dark noise. (C) Comparison of mean signals and HF noise floor between active and inactive fibers. Statistical significance was assessed by the Mann–Whitney U‐test after the Shapiro–Wilk normality test. Figure S4: Time‐frequency profile of photometry signals Top, normalized photometry signals. Bottom left, spectrogram of photometry signals. Bottom right, average power profile, normalized to percentage. Figure S5: Arousal state classification based on pupil diameter Top, representative pupil images. Bottom, the distribution of normalized pupil diameters. The 33 and 67 percentiles were the thresholds to classify arousal states. [file EJN-63-0-s003.docx]

**Supplementary Information**

Multi-fiber array-based photometry system for multi-regional functional mapping in the mouse brain

Manil Bradai*, Mirna Merkler*, Gabriela Gil, Rebecca Davie, Shuzo Sakata

Strathclyde Institute of Pharmacy and Biomedical Sciences, University of Strathclyde

* equal contributions

**Supplementary Table 1. Optical system parts list**

Please note that the price was based on March 2026.

**Supplementary Table 2. Surgical parameters**

**Supplementary Table 3. Parameters for data analysis**

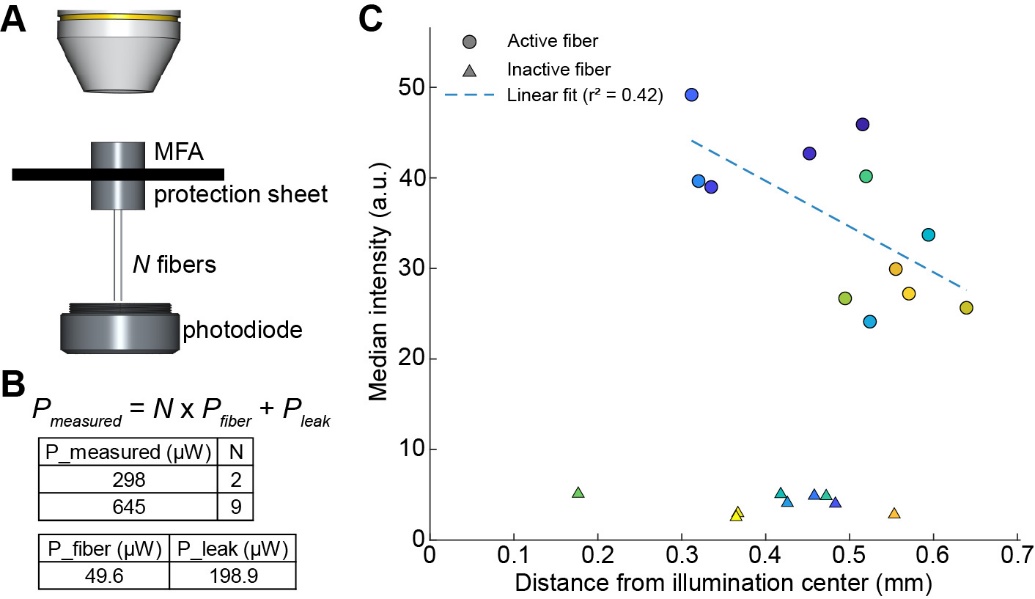


**Supplementary Figure 1. Light power output estimation.** (**A**) Diagram of an empirical approach to light output estimation. (**B**) A linear formula to estimate light output and the resultant tables. The light power from the objective lens was 69.3 mW. (**C**) The relationship between the modeled light power profiles and the distance from the illumination center. The values were calculated based on a quality control image (**Fig. 2D**). Pearson’s *r* = -0.65, *p* < 0.005.


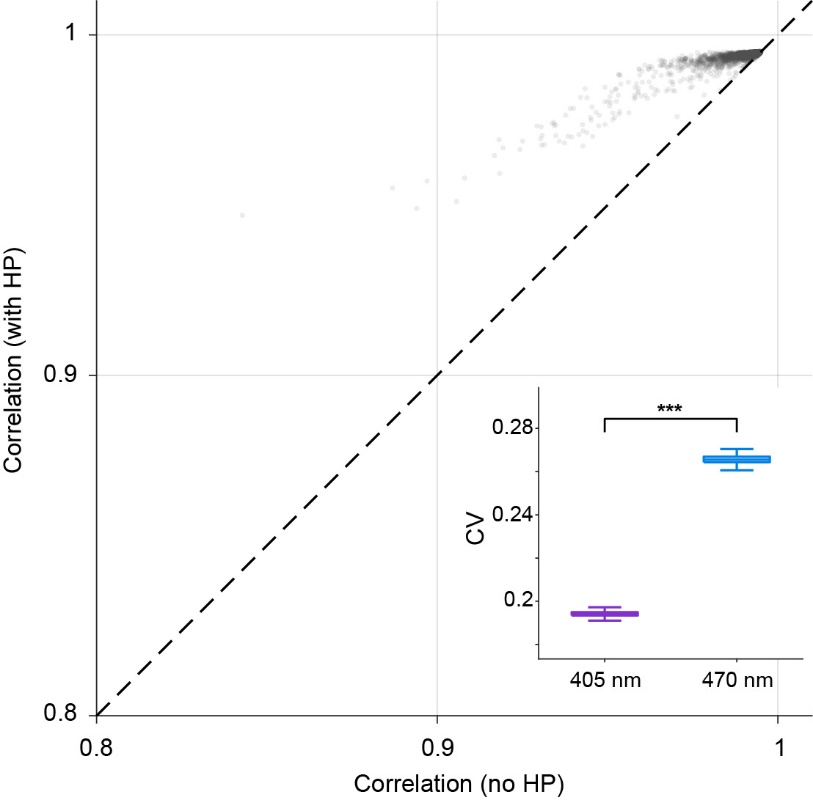


**Supplementary Figure 2.** The effect of preprocessing before motion correction on 405 nm images. Correlation to the reference was compared between two conditions, with and without a high-pass (HP) filter. *Inset*, Correlation of variation in pixel intensity. ***, *p* < 0.001, Wilcoxon signed-rank test.


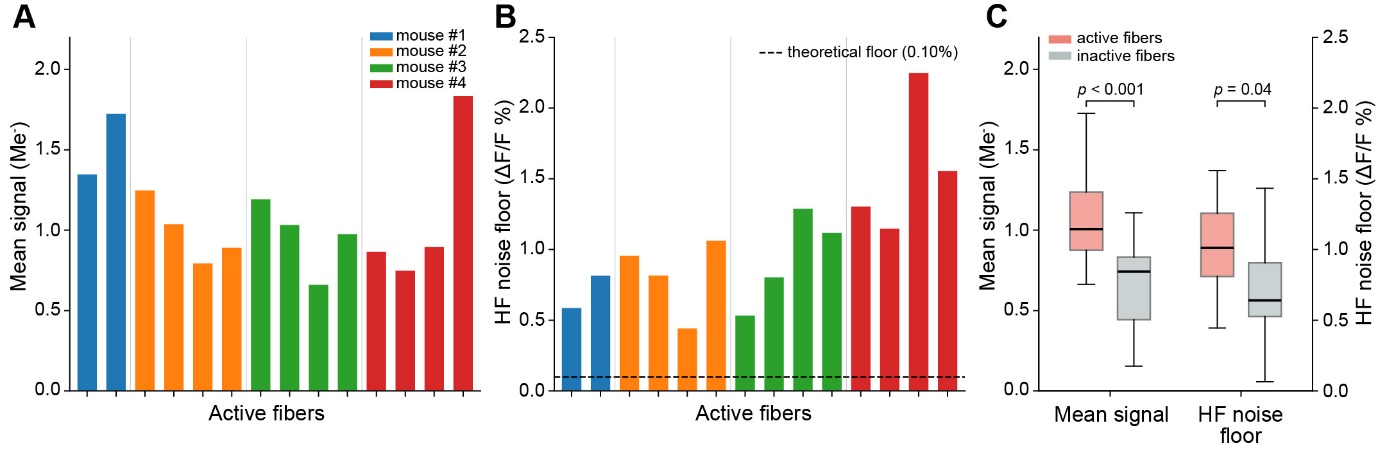


**Supplementary Figure 3. Noise budget analysis.** (**A**) Mean fluorescent signals in e^-^ across active fibers and experiments. (**B**) High-frequency (HF) noise floor across active fibers and experiments. The HF noise floor was computed using successive frame differences as std(ΔF)/√2, where ΔF is the first difference in the photoelectron time series. The HF noise floor was expressed in ΔF/F by normalizing them to the mean fluorescence. The total theoretical noise (dotted line) was estimated by estimating photon shot noise and camera read/dark noise. (**C**) Comparison of mean signals and HF noise floor between active and inactive fibers. Statistical significance was assessed by the Mann-Whitney U-test after the Shapiro-Wilk normality test.


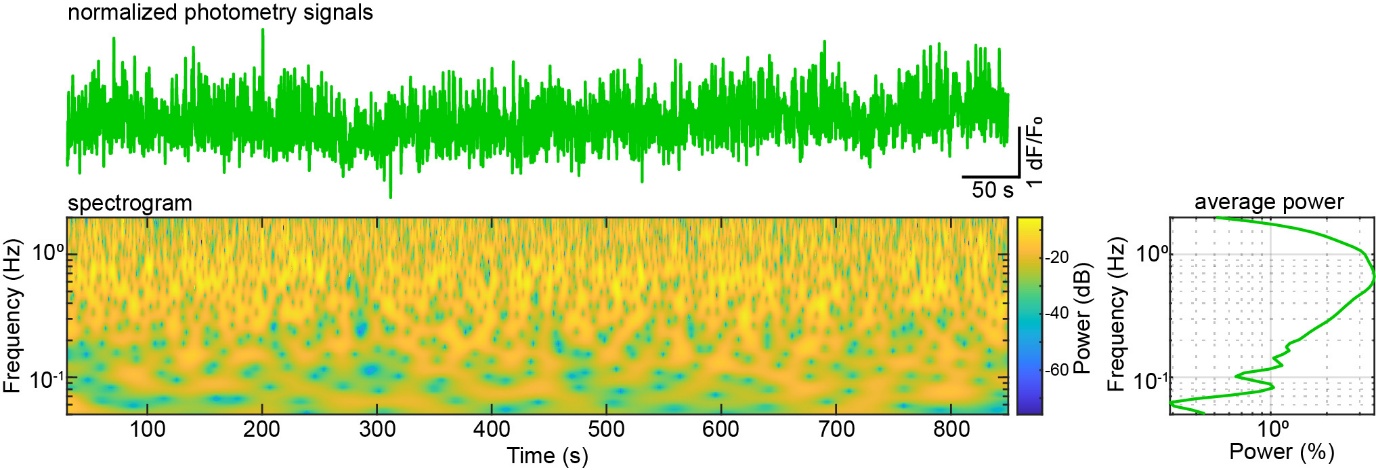


**Supplementary Figure 4. Time-frequency profile of photometry signals.** *Top*, normalized photometry signals. *Bottom left*, spectrogram of photometry signals. *Bottom right*, average power profile, normalized to percentage.


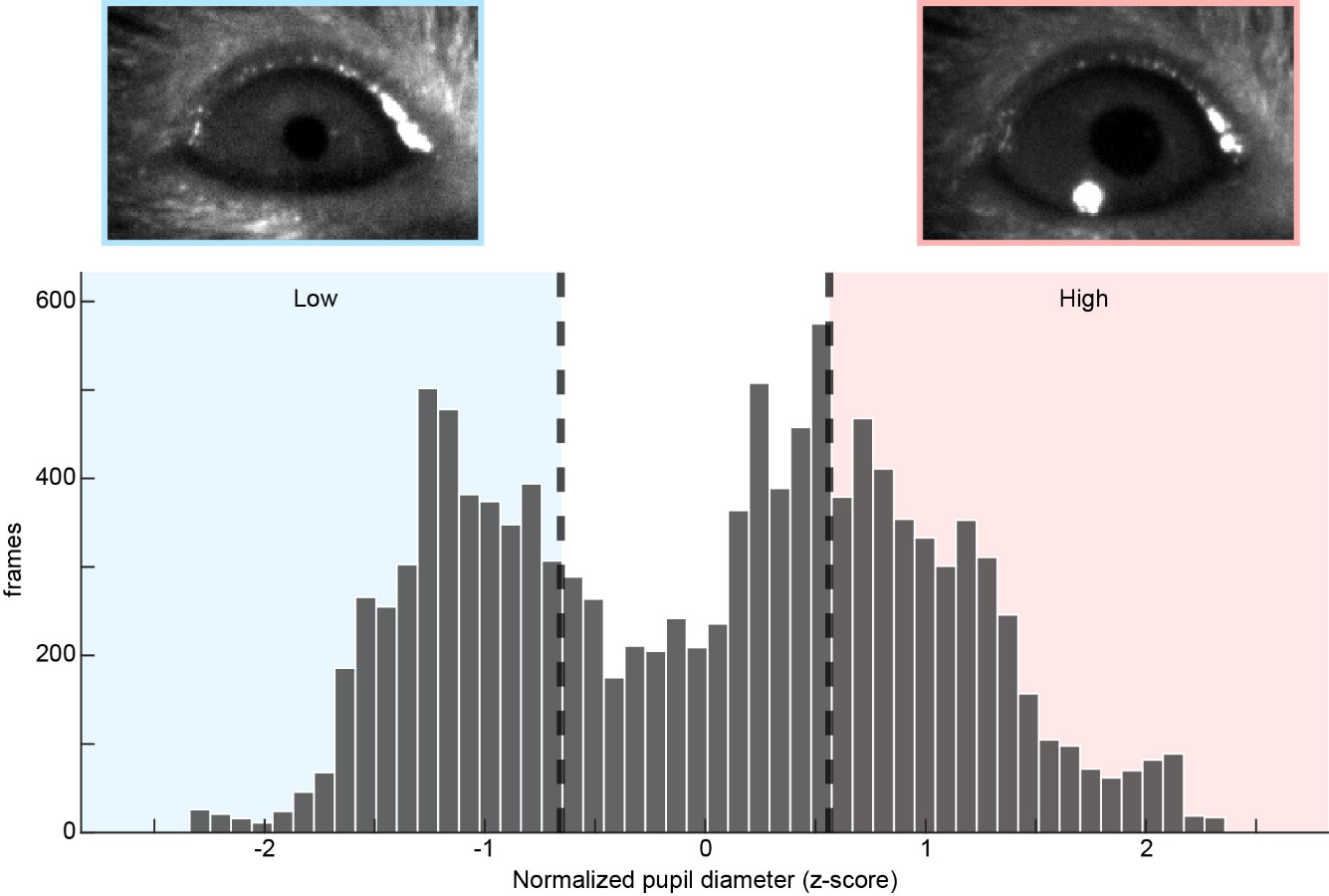


**Supplementary Figure 5. Arousal state classification based on pupil diameter**. *Top*, representative pupil images. *Bottom*, the distribution of normalized pupil diameters. 33 and 67 percentiles were the thresholds to classify arousal states.
