# Supplementary material for: Multifiber Array‐Based Photometry System for Multiregional Functional Mapping in the Mouse Brain
Source: Eur J Neurosci. 2026 Jun 17;63(12):e70582. doi: 10.1111/ejn.70582 (PMC13273707; doi:10.1111/ejn.70582)
Supplement: Supplementary file 2 — Data S1: Supporting Information. [file EJN-63-0-s002.pdf]

Supplementary Note 1
Grid fabrication guide for multi-fiber array photometry system

Sakata Lab

Introduction
For the multi-fiber array (MFA) photometry system (Bradai et al., *bioRxiv* 2025), 3D-printed grids are required to construct an MFA. The grid allows arranging multiple optical fibers to target multiple brain regions for various photometry experiments *in vivo*. In this document, we provide a manual to prepare grids with additional technical notes.

Workflow
1. Designing a grid in FreeCAD
2. Exporting a CAD file as .stl file
3. 3D printing with Projection Micro Stereolithography (PμSL) technology

1. Designing a grind in FreeCAD
- Open FreeCAD

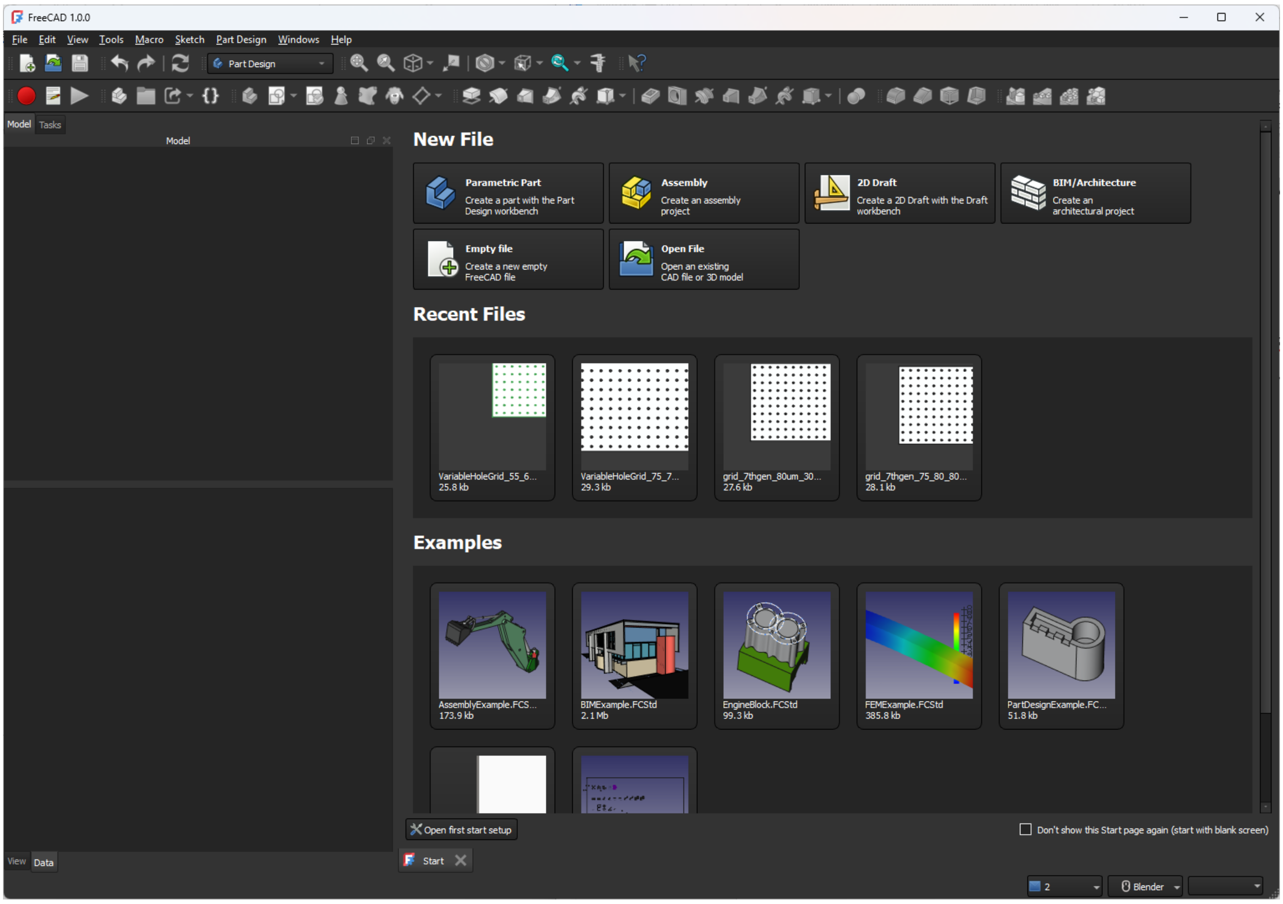

- File manu “Macro” > “Macros”

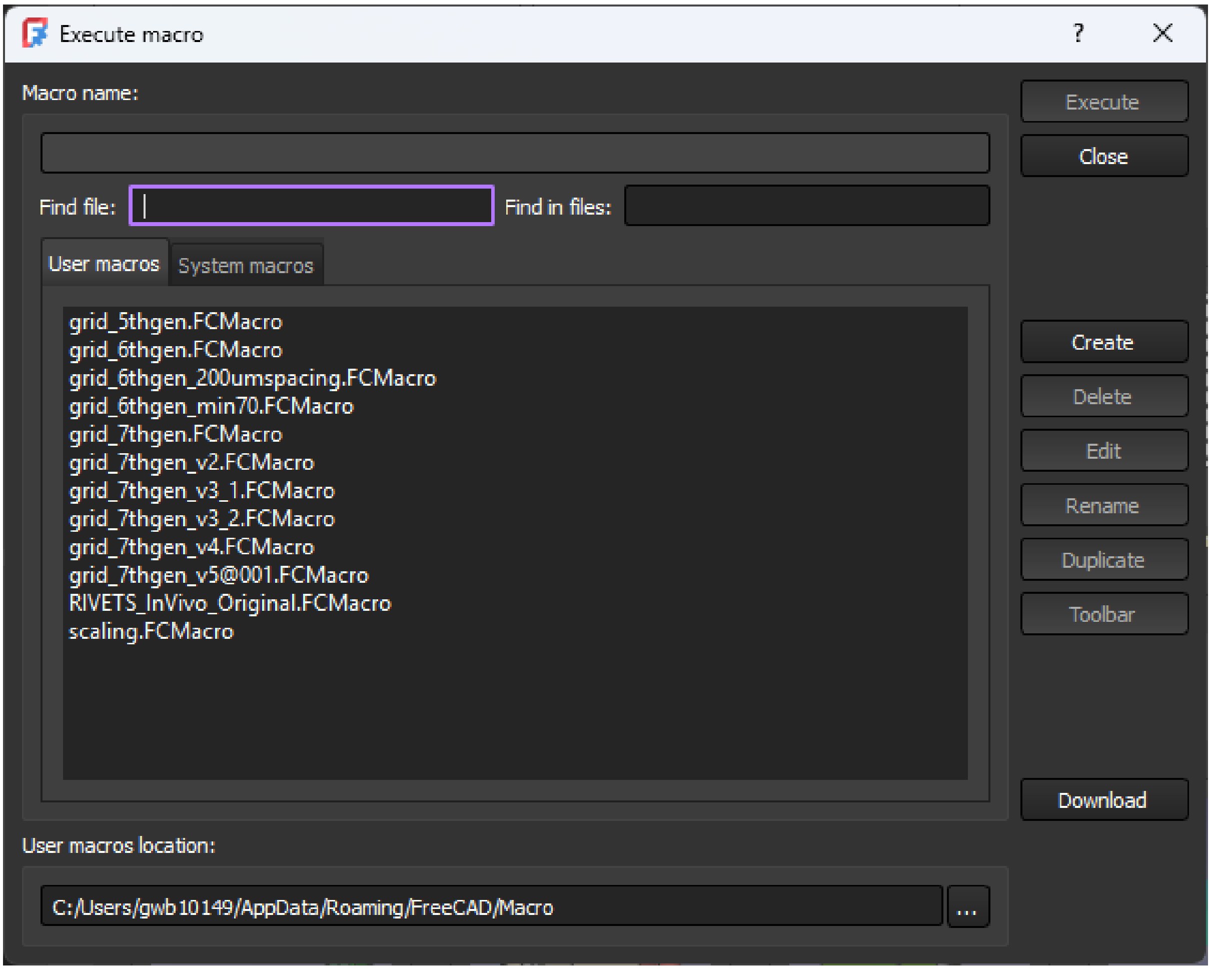

- Click “Create” (after naming the new macro, a Python editor will open)
- Copy and paste the following script:

```
import FreeCAD as App
import Part
import Mesh

doc = App.newDocument("my_grid_v1")

# --- Parameters ---
width = 4.0 # mm
height = 3.0 # mm
thickness = 0.5 # mm
spacing = 0.3 # mm ← 300 µm center-to-center
hole_sizes_um = [55, 60, 65] # µm 3 types of hole size

# --- Derived values ---
hole_sizes_mm = [d / 1000.0 for d in hole_sizes_um] # convert µm → mm
x_count = int(width / spacing)
y_count = int(height / spacing)
```

```
# --- Create base plate ---
plate = Part.makeBox(width, height, thickness)

# --- Generate holes with row-wise offset pattern ---
holes = []
for j in range(y_count):
for i in range(x_count):
idx = (i + j) % len(hole_sizes_mm) # shifts pattern each row
d = hole_sizes_mm[idx]
x = (i + 0.5) * spacing
y = (j + 0.5) * spacing
hole = Part.makeCylinder(d / 2, thickness, App.Vector(x, y, 0))
holes.append(hole)

# --- Cut holes from plate ---
for h in holes:
plate = plate.cut(h)

# --- Add to FreeCAD document ---
obj = doc.addObject("Part::Feature", "my_grid_v1")
obj.Shape = plate
doc.recompute()

print(f"✅ Grid created with {x_count}×{y_count} holes")
print(f" Hole diameters cycle through {hole_sizes_um} µm")
print(f" Hole spacing = {spacing*1000:.0f} µm")
print(f" Plate size = {width}×{height}×{thickness} mm³")

# --- Optional: export STL automatically ---
output_path = App.getUserAppDataDir() + "my_grid_v1.stl"
Mesh.export([obj], output_path)
print("💾 Exported STL to:", output_path)
```

- Save the Macro
- Macro > Macros … Select the created macro, then Execute

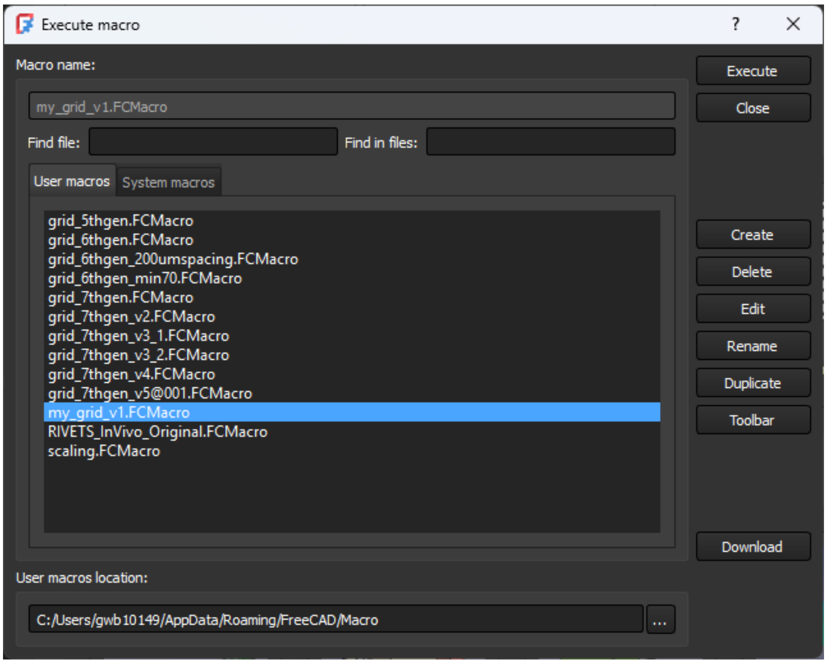

- A designed grid will be created

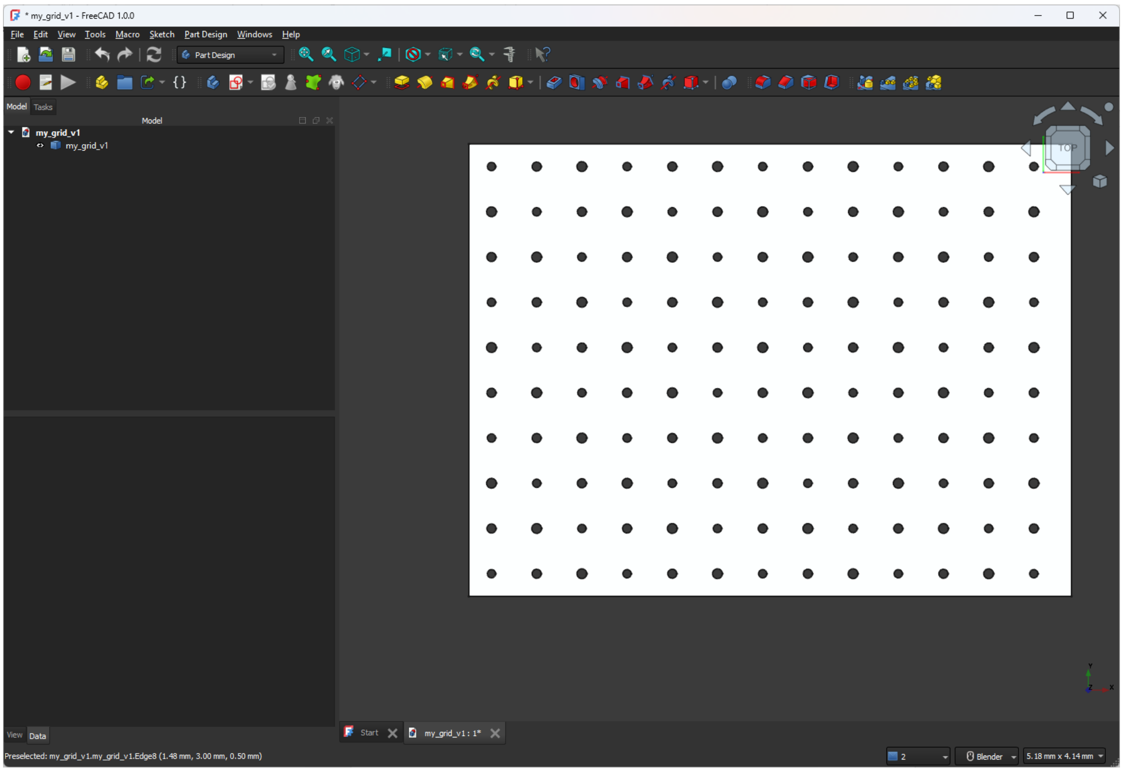

Technical note

The above script is a grid containing three different hole sizes (55, 60, 65 µm) with 0.3 mm spacing. The grid dimensions are 4 x 3 x 0.5 mm^3. By changing parameters in the Parameters section, any size of grid may be created.

2. Exporting a CAD file as .stl file

- Select the object (highlighted)

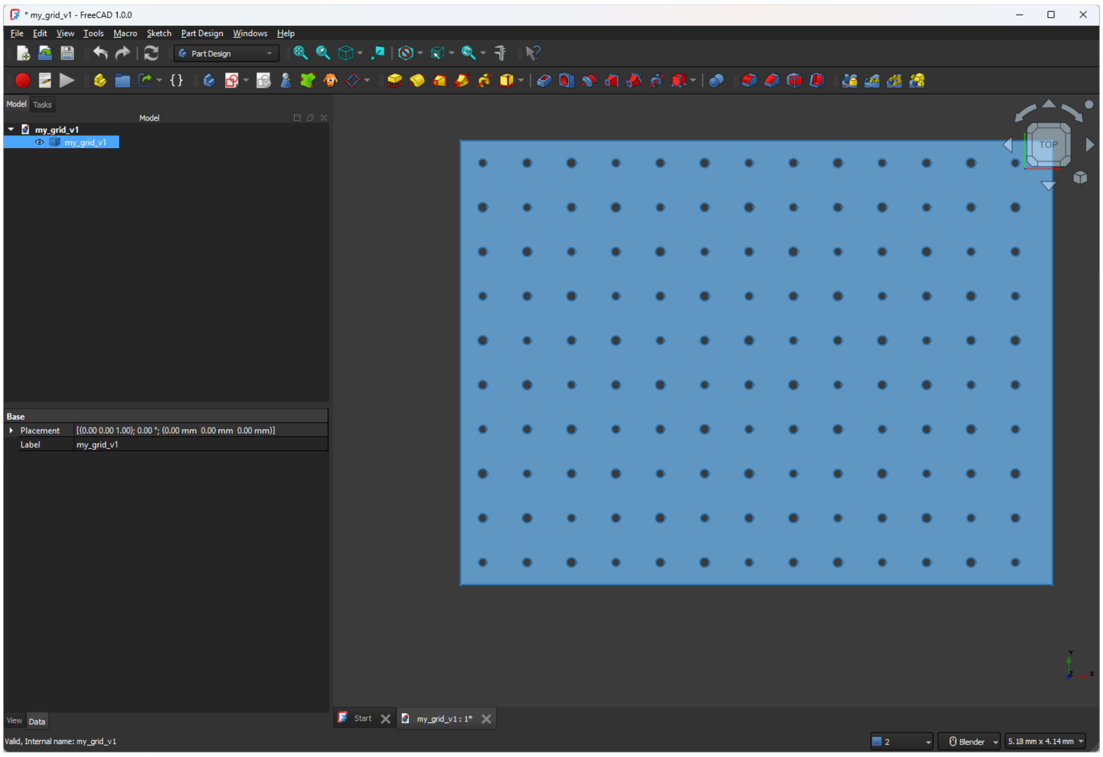

- File > Export …
- Export as .stl file

3. 3D printing with PμSL technology

- Contact the company capable of Project Micro Stereolithography (PμSL) technology
- Sending the exported .stl file, grids can be printed

Company information

- BMF https://bmf3d.com/
- IPFL, https://www.ipfl.co.uk/

Any companies using PμSL, provided by Boston Micro Fabrication (BMF), should be able to print grids.

Technical note

At least two factors may be consulted with a company: (1) materials and (2) resolution

- **Materials:** High temperature liquid (HTL) is standard, but transparent. For opacity, carbon-based materials may be considered.
- **Resolution:** 2 μm or 10 μm resolution printing is available. The cost difference may be approximately 3x.

When grids are printed with carbon-based materials with 2 μm resolution, a 65~70 μm hole in the CAD file may be recommended since the actual hole size will become ~60 μm, which is suitable for 50 μm optic fibers.
